# Bilateral proliferative retinopathy and ischemic optic neuropathy in a patient with atypical hemolytic-uremic syndrome: A case report

**DOI:** 10.1097/MD.0000000000017232

**Published:** 2019-09-27

**Authors:** I-Hung Lin, Ying-Jen Chen, Ping-Ying Chang, Po-Wei Hsiao, Tzu-Heng Weng, Yun-Hsiang Chang

**Affiliations:** a Department of Ophthalmology; b Division of Hematology/Oncology, Department of Internal Medicine, Tri-Service General Hospital, National Defense Medical Center; c School of Medicine of The National Defense Medical Center, Taipei City, Taiwan, ROC.

**Keywords:** hemolytic uremic syndrome, optic neuropathy, proliferative retinopathy, tractional retinal detachment, vitreous hemorrhage

## Abstract

**Rationale:**

To report a rare case of severe atypical hemolytic-uremic syndrome (HUS) in a patient who presented with vitreous hemorrhage and tractional retinal detachment (TRD) in both eyes. To our knowledge, this is the first reported case of atypical HUS complicated with bilateral TRD in the literature.

**Patient concerns:**

A 20-year-old man with atypical HUS demonstrated bilateral visual acuity of hand motion at 30 cm.

**Diagnoses:**

Dilated fundus examination revealed diffuse intraretinal hemorrhage with vascular engorgement, neovascularization of the disc, and neovascularization elsewhere bilaterally. Fluorescein angiography revealed bilateral proliferative retinopathy, retinal hemorrhage, and a large nonperfusion area with extensive neovascularization. Intravitreal antivascular endothelial growth factor (ranibizumab) injection was administered in both eyes, but his ophthalmic condition did not improve, and TRD developed bilaterally. Therefore atypical HUS complicated with bilateral TRD was diagnosed.

**Interventions:**

Pars plana vitrectomy was performed with panretinal photocoagulation and silicone oil tamponade in the right eye.

**Outcomes:**

After the pars plana vitrectomy of right eye, the retina was well-attached after surgery, but visual acuity remained poor. Visual evoked potential examination showed poor waveforms bilaterally, which suggested ischemic optic neuropathy.

**Lessons:**

Atypical HUS can cause systemic thrombotic microangiopathy, resulting in ischemic retinal changes. These ischemic retinal changes can then cause hypoxia, which triggers production of angiogenic factors and subsequently causes retinal vascular hyperpermeability, retinal and vitreous neovascularization, fibrovascular proliferation, vitreous hemorrhage, and TRD, in a manner similar to that of other ischemia-induced proliferative retinopathies. Despite successful surgery in the right eye, our patient's visual acuity did not improve, possibly because of severe and generalized ischemia of intraocular tissue, which resulted in ischemic optic neuropathy.

## 1 Introduction

Atypical hemolytic uremic syndrome (HUS) is a rare disease, with an estimated incidence of 2 cases per 1 million people in the USA.^[1]^ It is an inherited disease, caused by chronic, uncontrolled hyperactivation of the complement system, which leads to systemic thrombotic microangiopathy (ie, formation of clots in small blood vessels throughout the body). This results in a variety of complications, including myocardial infarction, kidney failure, and ischemic retinal changes. However, HUS typically exhibits no ophthalmic manifestations. A few cases of Purtscher-like retinopathy have been reported.^[2]^ Here, we report the case of a 20-year-old man with severe atypical HUS who presented with vitreous hemorrhage and tractional retinal detachment (TRD) in both eyes. To our knowledge, this is the first reported case of atypical HUS complicated with bilateral TRD.

## 2 Case report

A 20-year-old Asian man, who is a soldier, presented to our emergency room with fever, general malaise, and seizures with altered consciousness. He had a temperature of 38°C, a platelet count of 22,000/mL, blood urea nitrogen 46 mg/dL, creatinine 3.0 mg/dL, hemoglobin 8.9 g/dL, lactic dehydrogenase 2105 U/L, haptoglobin <5.8 mg/dL, and serum ADAMTS13 activity of 49.4%. Brain computed tomography and magnetic resonance imaging showed no intracranial lesion, intracerebral hemorrhage, or ischemic stroke. There was no diarrhea. Atypical HUS complicated by acute kidney injury and central nervous system involvement was diagnosed. He received plasma exchange with 24-U of fresh-frozen plasma daily for 2 weeks, 2-U packed red blood cell transfusions daily for 8 days, hemodialysis for 1 week, and a novel long-acting C5 complement inhibitor (ravulizumab) for 2 months. His altered consciousness, thrombocytopenia, anemia, and acute kidney injury improved gradually.

**Figure 1:**
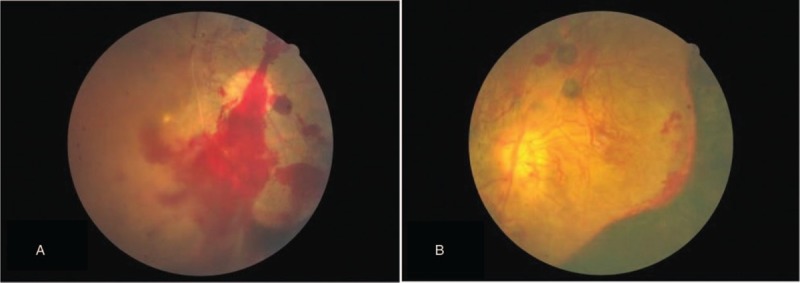
Fundus color photography of the patient's right eye (A) and left eye (B). Neovascularization and retinal and vitreous hemorrhage were noted in both eyes.

**Figure 2:**
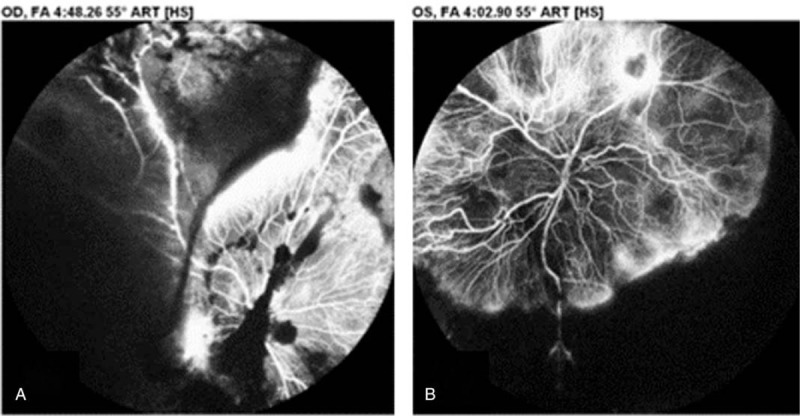
Fluorescein angiography of the patient's right eye (A) and left eye (B). Vitreous and retinal hemorrhage, as well as a large nonperfusion area with severe neovascularization, were noted in both eyes.

Approximately 2 months after his original presentation, his vision began to deteriorate in both eyes. His visual acuity was hand motion at 30 cm bilaterally. Intraocular pressure was 18 mm Hg on the right and 12 mm Hg on the left. Both anterior segments were unremarkable except that the pupils were mid-dilated with a sluggish light reflex. Dilated fundus examination revealed diffuse intraretinal hemorrhage with vascular engorgement, neovascularization of the disc, and neovascularization elsewhere bilaterally (Fig. 1). Fluorescein angiography revealed bilateral proliferative retinopathy, retinal hemorrhage, and a large nonperfusion area with extensive neovascularization (Fig. 2). An intravitreal anti-vascular endothelial growth factor (VEGF) (ranibizumab, Lucentis) injection was administered in both eyes.

**Figure 3:**
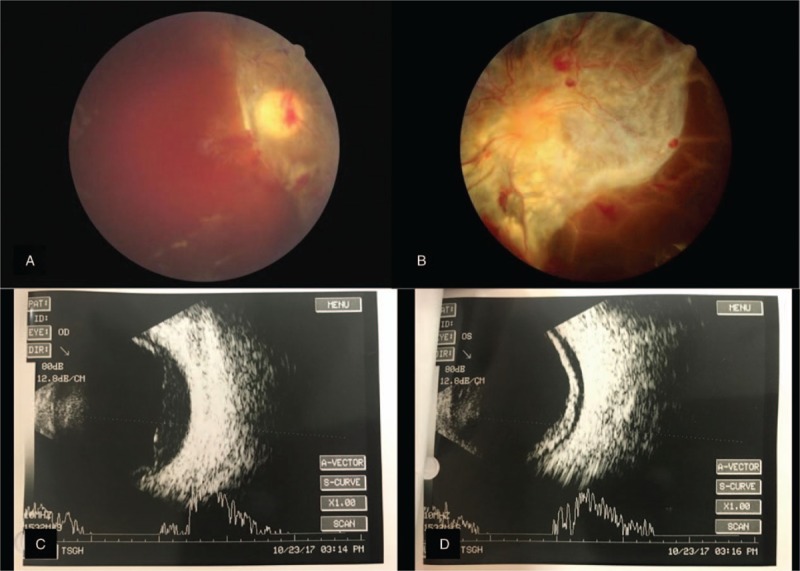
(A, B) Fundus photographs and (C, D) B-scan sonographic images of both eyes at 1 month after treatment with an intravitreal antivascular endothelial growth factor agent. Severe fibrovascular proliferation with hemorrhage and tractional retinal detachment are present in the right eye (A, C) and left eye (B, D).

**Figure 4:**
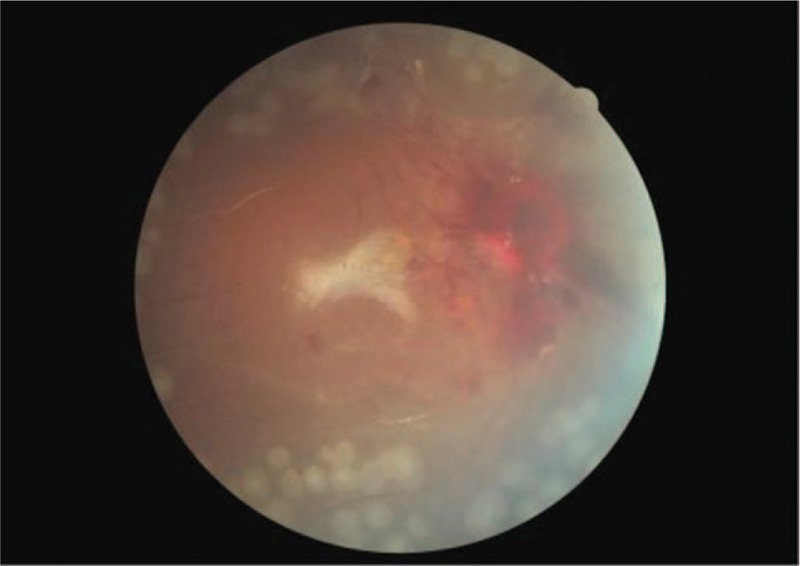
Fundus color photograph of the right eye after surgery. Although there is visible hemorrhage around the optic disc, the retina is well-attached.

**Figure 5:**
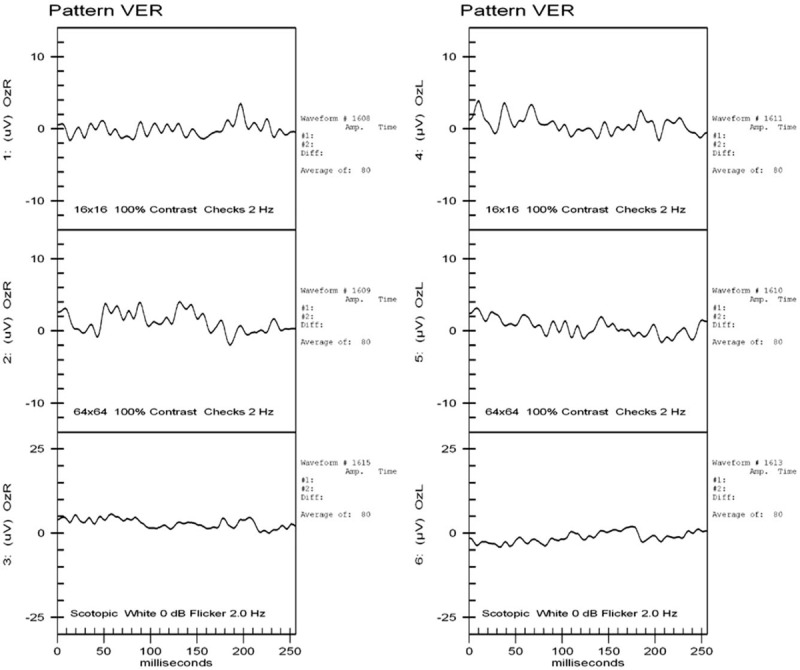
Visual evoked potential examination showed poor waveforms in both eyes.

His ophthalmic condition did not improve, and TRD developed bilaterally (Fig. 3). We performed pars plana vitrectomy with panretinal photocoagulation and silicone oil tamponade in the right eye approximately 3 months after his original presentation. The retina was well attached after surgery (Fig. 4), but the visual acuity did not improve. The visual evoked potential examination showed poor waveforms bilaterally (Fig. 5).

## 3 Discussion

HUS is a thrombotic microangiopathy characterized by microangiopathic hemolytic anemia, thrombocytopenia, and acute kidney failure. It can be classified as typical (with diarrhea) or atypical (without diarrhea). Typical HUS is often caused by Shiga toxin-producing *Escherichia coli* type 0157:H7.^[3]^ Thrombotic thrombocytopenic purpura (TTP) is also a similar disease of thrombotic microangiopathy, characterized by microangiopathic hemolytic anemia, severe thrombocytopenia, and microthrombi in several organs (eg, the heart, central nervous system, and, to a lesser degree, the kidneys).^[4]^ Serum ADAMTS13 is a zinc-containing metalloprotease enzyme that cleaves von Willebrand factor, a large protein involved in blood clotting. Its activity is often <10% in TTP, and can be used to distinguish between typical and atypical HUS; notably, atypical HUS often exhibits a high level of serum ADAMTS13.^[5]^ Herein, we have described a patient who had anemia, thrombocytopenia, and acute kidney failure, as well as absence of diarrhea, absence of *E coli* or other pathogens on stool culture, and serum ADAMTS13 activity of 49.4%. Therefore, atypical HUS was diagnosed. Two months after the diagnosis, the patient developed a very rare complication of severe bilateral proliferative retinopathy resulting in bilateral TRD and ischemic optic neuropathy.

Atypical HUS has a genetic basis and is caused by chronic, uncontrolled hyperactivation of the complement system,^[6]^ which leads to systemic thrombotic microangiopathy, that is, formation of clots in small blood vessels throughout the body, resulting in a variety of diseases, including myocardial infarction, kidney failure, and ischemic retinal changes.^[7]^

Ischemic retinal changes are rare in patients with atypical HUS. Sturm et al performed eye examinations in 69 of 87 children admitted with HUS over a 12-year period, including those without visual complaints, and found ocular involvement in only 3 patients; 2 of these patients had Purtscher-like presentations and 1 eventually developed neovascularization of the disc.^[2]^ Ischemic retinal changes can cause hypoxia, which triggers production of angiogenic factors, including insulin-like growth factor 1, basic fibroblast growth factor, erythropoietin, and VEGF. This may lead to retinal vascular hyperpermeability, retinal and vitreous neovascularization, fibrovascular proliferation, vitreous hemorrhage, and TRD, similar to other ischemia-induced proliferative retinopathies. Another possible source of TRD may be the “crunch” phenomenon: after anti-VEGF therapy, the response to reduced levels of VEGF may cause regression of new vessels and subsequent contraction of associated fibrovascular tissue, which causes the onset or worsening of TRD. This “crunch” phenomenon has also been found in proliferative diabetic retinopathy^[8]^ and retinopathy of prematurity.^[9]^ Early panretinal photocoagulation might prevent the progression and development of subsequent proliferative retinopathy and TRD in these patients.

To our knowledge, this is the first reported case of atypical HUS complicated by bilateral TRD. Sandhu et al reported a case of typical HUS with bilateral TRD in 2015.^[10]^ Serous retinal detachment in patients with HUS has also been reported, presumably secondary to choroidal ischemia.^[11]^ Even after successful surgery in the right eye, our patient's visual acuity was not improved, possibly because of very severe and generalized ischemia of the intraocular tissue, including the optic nerve, causing ischemic optic neuropathy.

In summary, HUS is a life-threatening condition that can involve multiple organs. Physicians should keep eye involvement and ocular symptoms in mind when examining a patient with HUS. Early treatment is essential to avoid vision-threatening complications.

## Author contributions

**Data curation:** I-Hung Lin, Po-Wei Hsiao.

**Investigation:** I-Hung Lin, Po-Wei Hsiao.

**Resources:** Tzu-Heng Weng.

**Writing – original draft:** I-Hung Lin.

**Writing – review and editing:** I-Hung Lin, Ying-Jen Chen, Ping-Ying Chang, Yun-Hsiang Chang.

I-Hung Lin orcid: 0000-0003-4268-2540.
